# O que a *Pesquisa Nacional de Saúde* tem a dizer sobre a influência do gasto com cigarro no rendimento domiciliar? 

**DOI:** 10.1590/0102-311XPT175423

**Published:** 2024-04-22

**Authors:** André Szklo, Mirian Carvalho de Souza, Aline de Mesquita Carvalho

**Affiliations:** 1 Instituto Nacional de Câncer José Alencar Gomes da Silva, Rio de Janeiro, Brasil.

**Keywords:** Política Pública de Saúde, Renda per Capita, Fumar Cigarros, Health Public Policy, Per Capita Income, Cigarette Smoking, Política Pública de Salud, Renta per Cápita, Fumar Cigarrillos

## Abstract

Em um Brasil no qual os indicadores de empobrecimento da população seguem aumentando, preocupa o fato de que indivíduos gastem dinheiro para comprar cigarro em vez de usarem esse recurso em ações que fortaleçam aspectos do bem-estar de suas jornadas de vida e de suas famílias. Estimou-se, a partir da *Pesquisa Nacional de Saúde* de 2019, a influência que o gasto com cigarro industrializado teve no orçamento familiar nos domicílios com pelo menos um fumante, estratificada por características sociodemográficas. Os fumantes brasileiros destinaram cerca de 8% do rendimento médio mensal domiciliar *per capita* para a compra de cigarros industrializados. O percentual do gasto médio mensal chegou a quase 10% desse rendimento, entre os fumantes de 15 a 24 anos, e foi ainda maior para aqueles com Ensino Fundamental incompleto (aproximadamente 11%). Nas regiões Norte e Nordeste do país, esse gasto ultrapassou os 9%. O estado com o maior comprometimento da renda domiciliar foi o Acre (13,6%), seguido por Alagoas (11,9%), Ceará, Pará e Tocantins (todos com aproximadamente 11%). Nossos achados reforçam, portanto, a importância de fortalecer a implementação de medidas efetivas de redução da proporção de fumantes, tal como a política tributária. Dessa forma, o dinheiro que atualmente é destinado pelos indivíduos à compra de cigarros poderá ser revertido no atendimento de suas necessidades básicas, contribuindo para a promoção da saúde e melhoria da qualidade de vida.

## Abstract (en)

In a country whose indicators of population impoverishment continue to increase, it is concerning that individuals spend money to buy cigarettes instead of using this resource in actions that strengthen aspects of the well-being of their lives and that of their families. Based on the *Brazilian National Health Survey* conducted in 2019, the influence of spending on manufactured cigarettes on the family budget in households with at least one smoker was estimated, stratified by sociodemographic characteristics. Brazilian smokers allocated around 8% of their average per capita monthly household income to the purchase of manufactured cigarettes. The percentage of average monthly expenditure on cigarettes reached almost 10% of this income among smokers aged 15 to 24 and was even higher for those with incomplete elementary education (approximately 11%). In the North and Northeast regions of the country, this expenditure exceeded 9%. The state with the most significant impact on household income was Acre (13.6%), followed by Alagoas (11.9%), Ceará, Pará, and Tocantins (all with approximately 11%). Our findings, therefore, reinforce the importance of strengthening the implementation of effective measures, such as tax policy, to reduce the proportion of smokers. Thus, the money that individuals currently allocate to purchase cigarettes can be used to meet their basic needs, contributing to the promotion of health and improving the quality of life.

## Abstract (es)

En un Brasil donde los indicadores de empobrecimiento de la población siguen aumentando, es preocupante el hecho de que las personas gasten dinero para comprar cigarrillo en lugar de usarlo en acciones para fortalecer los aspectos del bienestar de sus vidas y la de sus familias. A partir de la *Encuesta Nacional de Salud* brasileña realizada en 2019, se estimó la influencia del gasto en cigarrillo industrializado en el presupuesto familiar de los hogares donde vivía al menos un fumador, estratificado por características sociodemográficas. Los fumadores brasileños destinaron alrededor del 8% del ingreso per cápita mensual promedio del hogar para la compra de cigarrillos industrializados. El porcentaje del gasto mensual promedio en cigarrillos alcanzó casi el 10% de este ingreso entre los fumadores de 15 a 24 años y fue aún mayor para los que tenían educación primaria incompleta (aproximadamente el 11%). En el Norte y Nordeste del país, ese gasto superó el 9%. El estado con un mayor compromiso con los ingresos del hogar fue Acre (el 13,6%), seguido por Alagoas (el 11,9%), Ceará, Pará y Tocantins (todos con aproximadamente el 11%). Por lo tanto, nuestros resultados resaltan la importancia de fortalecer la implementación de medidas efectivas para reducir la proporción de fumadores, tal como la política tributaria. Así, el dinero que actualmente las personas destinan a la compra de cigarrillos podría utilizarse en la atención de sus necesidades básicas, contribuyendo a promover la salud y la mejora de la calidad de vida.

## Introdução

No Brasil, cerca de dois em cada três fumantes virão a falecer em decorrência desse comportamento de risco ^1^. De fato, já há evidência científica suficiente mostrando que os indivíduos que fumam têm um risco aumentado de mortalidade por todas as causas, quando comparado aos não fumantes, em função de uma piora no seu estado geral de saúde ^1^^,^^2^.

Dados do sistema de monitoramento da epidemia do tabagismo no Brasil apontam que essa epidemia está concentrada nas populações de renda e escolaridade mais baixas ^3^^,^^4^. E são justamente os indivíduos em piores condições socioeconômicas que apresentam os percentuais mais elevados de percepções dos seus estados de saúde como regular, ruim ou muito ruim, em comparação aos indivíduos menos vulneráveis ^4^.

Uma parte considerável dos R$ 125 bilhões gastos anualmente com o tratamento de doenças associadas ao tabagismo, em custos diretos ao sistema de saúde e indiretos para a sociedade, poderia ser evitada se os fumantes atuais parassem de fumar e os adolescentes não iniciassem no tabagismo ^2^. Em um Brasil no qual os indicadores de empobrecimento da população não param de aumentar ^5^, preocupa, ainda, o fato de que indivíduos gastem dinheiro para comprar cigarro em vez de usarem esse recurso em ações que fortaleçam aspectos do bem-estar de suas jornadas de vida e das suas famílias.

O objetivo deste artigo é, portanto, estimar, a partir dos dados de uma pesquisa de representatividade nacional, a influência que o gasto com cigarro industrializado tem no orçamento familiar dos domicílios de fumantes, total e estratificado por características sociodemográficas.

## Metodologia

Este artigo utiliza os dados sociodemográficos e de comportamento de fumar da *Pesquisa Nacional de Saúde* (PNS) conduzida em 2019 ^4^. Essa pesquisa foi realizada pelo Instituto Brasileiro de Geografia e Estatística (IBGE) com uso de uma amostra probabilística estratificada e ponderada com quatro etapas de seleção (municípios, setores censitários, domicílios e indivíduos com 15 anos ou mais). Apenas um indivíduo por domicílio (n = 90.846) foi selecionado aleatoriamente para responder perguntas sobre o uso de tabaco, e 11.386 indivíduos relataram fumar atualmente. Maiores detalhes sobre a metodologia podem ser observados em publicações recentes ^4^^,^^6^.

As três perguntas que definiram o gasto mensal com cigarro industrializado foram as seguintes:

(1) “Na última vez em que o(a) Sr(a) comprou cigarros para uso próprio, quantos cigarros comprou?”, sendo que as opções de resposta eram em “cigarros” (unidade), “maços” (cigarros por maços × número de maços), “pacotes” (cigarros por maços × número de maços × número de pacotes) e “nunca comprei cigarros para uso próprio”. Após excluir os fumantes que afirmaram nunca ter comprado cigarros para uso próprio (1,9%), perguntou-se:

(2) “No total, quanto o(a) Sr(a) pagou por essa compra?”. Com isso, conseguiu-se calcular o preço pago por cigarro na última compra. Considerando que mais de 90% dos fumantes de cigarros industrializados são fumantes diários ^4^ e, portanto, devem ser norteados pela fidelidade à marca consumida e pela utilização da compra direta como principal modalidade de obtenção de cigarro, obteve-se a informação relativa ao consumo médio diário de cigarros dos fumantes.

(3) “Em média, quantos cigarros industrializados o(a) Sr(a) fuma por dia ou por semana atualmente?” também foi usada para se chegar ao gasto mensal com cigarro industrializado. Para o cálculo do rendimento mensal domiciliar, somaram-se os rendimentos brutos mensais, de todos os trabalhos (formais e/ou informais), de aluguel, das transferências (aposentadoria/pensão pública ou privada, programas sociais, pensão alimentícia etc.), de aplicações financeiras, e os não monetários (p.ex.: valores estimados em trocas de produtos ou mercadorias) de todos os membros do domicílio. Posteriormente, realizou-se a divisão do rendimento mensal domiciliar pelo número de moradores e, assim, obteve-se o rendimento mensal domiciliar *per capita*. Para todos os cálculos descritos, foram excluídos os indivíduos cuja condição na unidade domiciliar era a de pensionista, empregado doméstico ou parente do empregado doméstico.

Informações sociodemográficas descritas na literatura como relacionadas ao uso do cigarro ^3^^,^^4^ foram utilizadas nas análises, tal como segue: (1) sexo; (2) idade, classificada em 15 a 24 anos, 25 a 59 anos e 60 anos ou mais; (3) escolaridade, dividida em quatro categorias: menor do que o Ensino Fundamental completo, Ensino Médio incompleto, Ensino Médio completo e maior do que Ensino Médio completo; (4) orientação sexual, classificada em heterossexual e homossexual/bissexual; e (5) região de residência (Norte, Nordeste, Centro-oeste, Sudeste e Sul).

Estimou-se, inicialmente, o gasto médio mensal com cigarro industrializado para o Brasil e estratificado por sexo, idade, escolaridade e regiões do país. Ademais, para todos os domicílios em que pelo menos um dos moradores era um fumante que comprava seus próprios cigarros industrializados, estimou-se o rendimento médio mensal domiciliar *per capita* para as categorias das características sociodemográficas estudadas. Finalmente, estimou-se a proporção que o gasto com cigarro representava no rendimento *per capita* e seus respectivos intervalos de 95% de confiança (IC95%), a partir da estimativa da razão entre o gasto médio mensal com cigarro industrializado e o rendimento médio mensal domiciliar *per capita*. Todas as análises foram realizadas com o programa Stata, versão 15.0 (https://www.stata.com), considerando os pesos amostrais dos indivíduos.

## Resultados

Em 2019, os brasileiros que fumavam cigarros industrializados gastaram em média 7,8% do rendimento mensal *per capita* do domicílio em que viviam para comprar cigarros (Tabela 1). As maiores contribuições do gasto mensal com cigarro no orçamento das famílias, a partir da não sobreposição dos respectivos IC95%, foram observadas entre os homens (8,2%), adolescentes e jovens adultos (9,6%), indivíduos com Ensino Médio incompleto ou menos (10,7%), heterossexuais (7,9%) e moradores das regiões Norte (9,8%) e Nordeste (9,2%) do Brasil (*versus* as respectivas categorias de comparação).

**Tabela 1:** Proporção do gasto médio mensal dos fumantes para compra de cigarros industrializados relativo ao rendimento médio mensal domiciliar *per capita*, segundo características sociodemográficas. Indivíduos com idade ≥ 15 anos. *Pesquisa Nacional de Saúde*, Brasil, 2019.

Características sociodemográficas	Rendimento médio mensal domiciliar *per capita* (A) * [R$]	Gasto médio mensal com cigarro industrializado (B) ** [R$]	**Proporção do gasto com cigarro sobre o rendimento domiciliar *per capita* (A/B)** **[% (IC95%)]**
Total	1.378,17	107,28	7,8 (7,4-8,2)
Sexo			
Masculino	1.404,38	114,98	8,2 (7,8-8,6)
Feminino	1.342,26	96,81	7,2 (6,8-7,6)
Faixa-etária (anos)			
15-24	871,08	83,71	9,6 (8,3-11,0)
25-59	1.359,54	104,30	7,6 (7,1-8,1)
60 ou mais	2.023,79	116,64	5,8 (5,1-6,4)
Escolaridade			
< Ensino Fundamental completo	868,95	94,78	10,9 (10,2-11,6)
< Ensino Médio completo	983,74	102,11	10,4 (9,5-11,3)
Ensino Médio completo	1.447,03	122,21	8,4 (7,7-9,2)
> Ensino Médio completo	3.460,83	128,61	3,7 (3,3-4,1)
Orientação sexual ***			
Heterossexual	1.370,80	108,30	7,9 (7,5-8,3)
Homossexual ou bissexual	1.864,90	88,80	4,8 (3,4-6,1)
Região			
Norte	869,62	85,52	9,8 (8,5-11,2)
Nordeste	896,45	82,32	9,2 (8,3-10,0)
Centro-oeste	1.379,63	101,39	7,4 (6,5-8,1)
Sudeste	1.575,89	117,38	7,5 (6,8-8,1)
Sul	1.510,90	115,60	7,7 (7,2-8,0)

IC95%: intervalo de 95% de confiança.

Nota: todas as análises foram realizadas considerando os pesos amostrais dos indivíduos.

* Rendimento médio mensal domiciliar (R$) que inclua pelo menos um fumante (exclusive o rendimento das pessoas cuja condição na unidade domiciliar era pensionista, empregado doméstico ou parente do empregado doméstico) dividido pelo número de moradores no domicílio;

** Calculado a partir do preço pago (R$) pelo cigarro na última compra multiplicado pelo consumo médio mensal de cigarros;

*** 2,6% dos fumantes de cigarro industrializado foram excluídos da análise pois não sabiam a sua orientação sexual ou se recusaram a responder.

**Figura 1:**
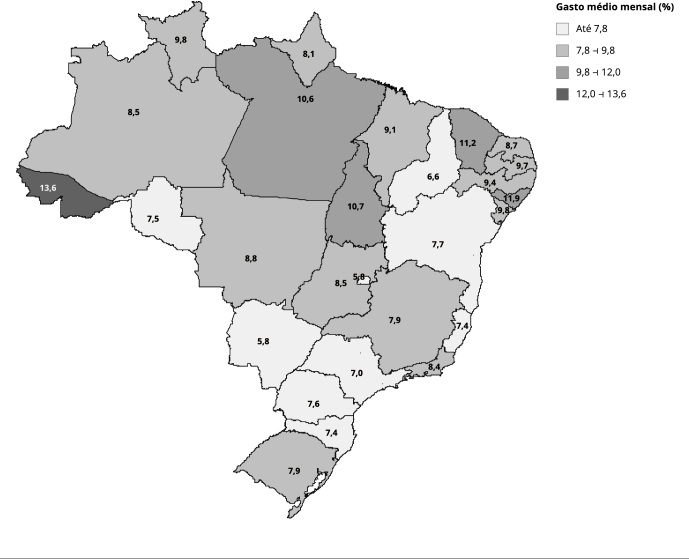
Gasto médio mensal com cigarros industrializados proporcional ao rendimento médio mensal domiciliar *per capita*, entre fumantes de cigarros industrializados, segundo Unidade da Federação. Indivíduos com idade ≥ 15 anos. *Pesquisa Nacional de Saúde*, Brasil, 2019.

A Figura 1 mostra a distribuição do percentual do gasto médio mensal com cigarro sobre o rendimento médio mensal domiciliar *per capita* entre os estados da Federação. Aquele com o maior comprometimento da renda domiciliar foi o Acre (13,6%), seguido por Alagoas (11,9%), Ceará (11,2%), Pará (10,6%) e Tocantins (10,7%). O estado que apresentou o menor comprometimento relativo do gasto com cigarro sobre o rendimento domiciliar *per capita* foi Mato Grosso do Sul (5,8%).

## Discussão

Cerca de 8% do rendimento médio mensal domiciliar *per capita* foi destinado, em 2019, para a compra de cigarros industrializados. O percentual do gasto médio mensal com cigarro chegou a quase 10% desse rendimento entre os fumantes de 15 a 24 anos e foi ainda maior para aqueles com Ensino Fundamental incompleto (aproximadamente 11%).

Os estados brasileiros com maior contribuição de gasto com cigarros estão localizados nas duas regiões com menor rendimento médio mensal domiciliar (Norte e Nordeste) ^4^. Não por acaso, o tabagismo está estreitamente relacionado à pobreza ^7^, fazendo com que o dinheiro que hoje é gasto para comprar cigarros não seja usado, por exemplo, com alimentação e educação. Além do impacto negativo econômico advindo de gastos e perda de produtividade causados pelas doenças e mortes associadas ao tabaco ^1^^,^^2^^,^^5^^,^^7^.

Os elevados gastos com cigarro refletem os desafios atuais existentes na implementação das ações da Política Nacional de Controle do Tabaco (PNCT), voltadas para a redução da iniciação e estímulo à cessação do fumo ^8^^,^^9^. A ausência de reajuste, desde 2016, das alíquotas do imposto que incide sobre os cigarros e do preço mínimo estabelecido por lei, aliada à forte presença de cigarros ainda mais baratos de origem ilegal ^8^, enfraquece uma das principais medidas (i.e., a política de preços e impostos) para reduzir a proporção de fumantes ^8^^,^^9^, principalmente entre os indivíduos mais desprovidos do ponto de vista socioeconômico. A regulamentação do imposto seletivo, de competência federal, sobre bens e serviços prejudiciais à saúde e ao meio ambiente, previsto pela reforma tributária aprovada no Congresso Nacional em 2023, representa, portanto, uma oportunidade única para voltar a aumentar a carga tributária sobre os cigarros industrializados e, consequentemente, reduzir o número de seus usuários.

A interferência da indústria do tabaco sobre a PNCT contribui não somente para essa realidade do preço baixo do cigarro ^10^, mas também para permitir que existam, no mercado brasileiro, cigarros com aromas e sabores que favorecem a iniciação ao fumo ^9^^,^^10^. Soma-se a isso o marketing, frequentemente ilegal, nos pontos de venda ^8^^,^^9^^,^^10^ e a baixa implementação da lei que proíbe a venda de cigarros para menores ^11^. O jovem brasileiro acaba sendo, portanto, uma vítima nessa cadeia de perpetuação do lucro da indústria da nicotina ao ser “capturado” para substituir uma parcela significativa dos fumantes atuais que virão a falecer ou deixarão de fumar em função, por exemplo, do surgimento de agravos de saúde ^1^^,^^2^.

Uma limitação inerente deste estudo está relacionada às informações de rendimento, consumo de cigarro e preço pago terem sido autorreferidas. Além disso, não é possível saber, de fato, a parcela do rendimento médio mensal domiciliar efetivamente alocada para cada residente. Ao se usar, contudo, o rendimento domiciliar *per capita*, conseguiu-se, pelo menos, ajustar pelo número de indivíduos residentes, o qual está relacionado positivamente com o fato de viver no Norte ou Nordeste do país, ter menor escolaridade e/ou ter um jovem fumante em casa ^4^; essa estratégia também contornou o fato de que não se dispunha da informação sobre a quantidade de fumantes em cada domicílio.

## Conclusão

Os achados reforçam, portanto, a importância de fortalecer a implementação de medidas efetivas de redução da proporção de fumantes, tal como a política tributária. Dessa forma, o dinheiro que atualmente é destinado pelos indivíduos à compra de cigarros poderá ser revertido no atendimento das suas necessidades básicas, contribuindo para a promoção da saúde e melhoria da qualidade de vida.
